# Priapism Got You Down? A Novel Method to Build Your Own Moderate Fidelity Task Trainer

**DOI:** 10.5070/M5.52240

**Published:** 2026-10-31

**Authors:** Malia J Moore, Rachell Laughlin, Dylan D Cooper

**Affiliations:** * Indiana University School of Medicine, Department of Emergency Medicine, Indianapolis, IN; ^ Indiana University Health, Indianapolis, IN

## Abstract

**Audience:**

This task trainer was designed to instruct emergency medicine (EM) residents and faculty at all levels of training.

**Introduction:**

Priapism management is time-sensitive, and the clinical opportunities to perform dorsal penile nerve block and corporal aspiration are seldom practiced. Despite knowledge of the importance of prompt recognition and management of priapism, many emergency physicians may not actually perform the recommended procedures to manage it for a variety of reasons.^1^ Due to the sensitive nature of the condition, physicians are eager to learn about and practice these intimate maneuvers in the safe setting of simulation; however, there is not a commercial task trainer available.^2^ Some low-fidelity creations have been published as solutions, but they are less realistic and time consuming to make, limiting feasibility and usability.^3^–^7^ One published creation inspired this task trainer, but the main structure is no longer available to be purchased, underscoring the need for a model that can be reproduced and recreated by the educator with flexible starting materials.^3^ Others are more time-consuming to make and require specific simulation equipment^4^ or may not be easily reused.^5^, ^6^ Other published low-fidelity creations are less anatomically accurate, limiting the fidelity of practice regarding the most intimate aspects of the procedure.^7^, ^8^

**Educational Objectives:**

By the end of this educational session, participants should be able to: 1) identify indications, contraindications, and potential complications of priapism management, 2) gather appropriate supplies and describe relevant landmarks, anatomy, and patient positioning for management of priapism, 3) demonstrate a dorsal penile nerve block, aspiration of corpus cavernosum, and intracavernosal phenylephrine administration to treat priapism, and 4) develop an approach to manage common pitfalls of priapism care.

**Educational Methods:**

We developed a more realistic and replicable priapism task trainer using materials readily accessible in a simulation lab. Emergency medicine faculty and simulation center staff collaborated to create a task trainer constructed from silicone and internal tubing, using the mold of a phallic-shaped candle (see attachment). This model was then used in a 30-minute training session, which included ten minutes of interactive discussion reviewing the steps of dorsal penile block and priapism management with PowerPoint images as a visual aid. Next, learners performed the procedure from start to finish with faculty guidance, followed by an opportunity to ask questions. The training session was open to EM residents, pediatric emergency medicine (PEM) fellows, EM community physicians, and EM academic faculty.

**Research Methods:**

This study was approved as exempt status by the Indiana University Institutional Review Board. Following the educational session, participants completed a voluntary survey evaluating the task trainer’s realism, its usefulness as a teaching tool, and their change in confidence in performing priapism management.

**Results:**

The mixed group included 14 EM residents, five PEM fellows, four EM community physicians, and four EM academic physicians. Of 27 participants who used the task trainer, the majority (92.6%) of survey respondents agreed or strongly agreed that the task trainer was realistic. Learners reported increased confidence in knowledge of the procedural steps and increased confidence in potentially performing the procedure in the clinical setting (Table 1). Most participants would recommend the training to a provider of a similar level of training.

**Discussion:**

Overall, the educational content was effective since learners reported an increase in knowledge of the steps of the procedure in addition to improved confidence to perform the procedure safely and successfully in the clinical environment. While many learners had never performed this procedure before in the clinical setting, the majority would recommend this training to a colleague of the same level of training. While the need for training in this procedure is clear,^1^–^2^ the options for available task trainers have remained limited. There is no commercial priapism task trainer, and published creations have varied strengths and weaknesses in balancing fidelity, cost-effectiveness, reproducibility, and reusability.^3^–^7^ The main takeaway from this training is that a realistic, durable, and low-cost task trainer can be developed to effectively teach how to treat a rare and sensitive procedure.

**Topics:**

Priapism, penile nerve block, dorsal nerve block, urology, aspiration, decompression.

## USER GUIDE

**Table:** 

List of Resources:	
Abstract	9
User Guide	11
Appendix A: Priapism Got You Down? PowerPoint	17

**Learner Audience:**

This task trainer was designed to instruct emergency medicine (EM) residents and faculty at all levels of training.

**Time Required for Implementation:**

Instructors will need to dedicate two hours overall for preparation, with one-hour hands-on dedicated time and another hour for the silicone to cure. This innovation has been used in five sessions with over 40 learners without degrading. Tubing leaks may eventually occur and can be patched as needed, or the model replaced.

Learners can develop this skill in a relatively short amount of time, with 15 minutes of small group discussion and 5–10 minutes of individual hands-on simulation of the procedure.

**Recommended Number of Learners per Instructor:**

We suggest small groups of 3–6 learners per instructor.

**Topics:**

Priapism, penile nerve block, dorsal nerve block, urology, aspiration, decompression.

**Objectives:**

By the end of this educational session, participants should be able to:

1. Identify indications, contraindications, and potential complications of priapism management.
2. Gather appropriate supplies and describe relevant landmarks, anatomy, and patient positioning for management of priapism.
3. Demonstrate a dorsal penile nerve block, aspiration of corpus cavernosum, and instillation of phenylephrine to treat priapism.
4. Develop an approach to manage common pitfalls of priapism care.

### Linked objectives, methods and results

During an interactive didactic instruction with PowerPoint, participants will discuss anatomy, steps of the procedure, and appropriate application of the procedure. The instructor will ensure learners understand how to diagnose priapism and set up for and perform the procedure of corpora aspiration (objective 1), after which learners participate in a hands-on session with the task trainers (objective 2 and 3). Teaching this session as a simulation allows learners to practice with hands-on experience and develop muscle memory for technique practice. Real time simulation in small groups allows learners to not only practice the procedure of penile block priapism drainage but also provides an opportunity for them to receive feedback on performing genitourinary exams and appropriate sterile draping. Additionally, learners will be practicing the aforementioned objectives in an environment conducive to real time feedback and redirection to build confidence (objective 2). Participants will have an interactive discussion to review penile anatomy, procedure steps set-up, and appropriate completion of a penile nerve block and priapism drainage. This is followed by a hands-on simulation with the modified task trainer. Instructors will coach learners simultaneously and answer any questions (objective 3).

### Recommended pre-reading for instructor

1. Bivalacqua TJ, Allen BK, Brock GB, et al. The diagnosis and management of recurrent ischemic priapism, priapism in sickle cell patients, and non-ischemic priapism: An AUA/SMSNA Guideline. *J Urol*. 2022;208(1):43–52. doi:10.1097/JU.0000000000002767
2. Davis JE, Silverman MA. Urologic Procedures. In: *Roberts and Hedges’ Clinical Procedures in Emergency Medicine and Acute Care.* 7th ed. Philadelphia, PA: Elsevier; 2019:1141–1185.^13^
3. Dai JC, Ahn JS, Cannon ST, et al. Acute ischemic priapism management: An educational and simulation curriculum. *MedEdPORTAL*. 2018;14:10731. Published 2018 Jul 13. doi:10.15766/mep_2374-8265.10731^8^
4. Hootman J, Chang A, Alvarez A. ALIEM tricks of the trade. https://www.aliem.com/trick-of-tradeangiocatheter-manual-aspiration-priapism/ 2020. Accessed 1 August 2024. 14
5. Kadioglu A, Kadihasanoglu M, Muneer A, et al. Priapism: recommendations from the Fifth International Consultation on Sexual Medicine (ICSM 2024). *Sex Med Rev*. 2026;14(1):qeaf072.^15^

### Learner responsible content (LRC)

We did not assign our learners any content prior to the session; however, suggested readings, if desired, include:

1. Hootman J, Chang A, Alvarez A. ALIEM tricks of the trade. Accessed 1 August 2024.^14^ https://www.aliem.com/trick-of-trade-angiocatheter-manual-aspiration-priapism/ 2020
2. Kadioglu A, Kadihasanoglu M, Muneer A, et al. Priapism: recommendations from the Fifth International Consultation on Sexual Medicine (ICSM 2024). *Sex Med Rev*. 2026;14(1):qeaf072.^15^ doi: 10.1093/sxmrev/qeaf072
3. Silberman M, Hu EW, Leslie SW, et al. Priapism. [Updated 2023 May 30]. In: StatPearls [Internet]. Treasure Island (FL): StatPearls Publishing; 2024.^16^ https://www.statpearls.uk/point-of-care/27713

### Implementation Methods

This session can be run in a relatively brief time frame of 30 minutes for a group of three to six learners. We conducted our session on an educational/conference day in combination with other procedural training wherein groups rotated every 30 minutes. Upon arrival to the small group, the facilitator will begin by asking learners if they have ever encountered priapism in the clinical setting (to quickly gather a needs assessment). They can begin by discussing ischemic versus non-ischemic priapism, causes of priapism, anatomy, and general approach to treatment. This discussion should take less than five minutes with the instructor posing questions to the group and filling in content deficits as needed. Then, a volunteer should be asked to gather materials from a supplies table with assistance from their peers as needed. This table should include personal protective equipment (PPE), drape or surgical towels, cleaning solution such as betadine or chlorhexidine, syringes of varying size, needles of varying type and size (18-G, butterfly, angiocath), simulated medications of lidocaine and phenylephrine.

Once the learner has selected supplies with feedback from peers and instructor, they may perform a dorsal penile nerve block with feedback from instructor. Following this, they can perform aspiration of the corpus cavernosa. Learners should be encouraged to trial different angles and insertion sites for needles, apply drapes, and discuss where to stand while completing the procedure. They may also discuss how to instill phenylephrine and practice this technique as needed. The American Urologic Association 2022 updated guidelines suggest phenylephrine 100–500 mcg doses suspended in 1 ml of normal saline. This dose is optimally premixed by pharmacy to minimize risks of miscalculation or overdose. This may be administered every 5 minutes for up to 1 hour. These doses are given intra-cavernosally in the 3 or 9 o’clock position near the base of the penile shaft. American Urological Association guidelines recommend that aspiration should precede phenylephrine to permit oxygenated blood to fill the corpora and potentially improve the effect of phenylephrine.^9^ The session should remain interactive with the involvement of the whole group. Following the initial performance, each learner should individually perform the procedure with both positive and corrective feedback given by the instructor. While learners are performing the procedure, there may also be an ongoing discussion of pearls, pitfalls, and scenarios where consultation of urology is prudent.[Fig f1-jetem-11-4-i9]

### List of items required to replicate this innovation

1. Dragon Skin FX Pro- Part A and B purchased from Smooth On or third-party company (Amazon.com: Smooth-On Dragon Skin™ 20 Mold Making & Special Effects Silicone Rubber - Trial Unit: Arts, Crafts & Sewing)
2. Silc Pig or another Silicone Pigment (Smooth-On Silc Pig Color Sampler | BLICK Art Materials)
3. PIV Arm Tubing *(*Replacement Veins for Multipurpose Venous Training Arm from Nasco | AED Superstore - SB50849U)
4. Chest Drain Tubing *(*ConvaTec Night Drainage Container Set - 027062, 027060 | Vitality Medical)
5. Bachelorette Candle Mold (3D Printed or found on Etsy 3D Penis Silicone Mold, genital Shape Candle Mold, 3 Size Penis Resin Mold-adult Themed Candle Mold-dick Soap Mold-penis Fondant Mold - Etsy
6. Mold Release Spray (Ease Release 200 Spray - 12 oz | BLICK Art Materials)
7. Other supplies include: rubber bands, tongue depressors, paint brush, binder clips, paper or plastic cups, gloves.

### Approximate cost of items to create this innovation

Materials may be purchased for $100–$150 if not readily available. Once materials are purchased (and depending on amount purchased), multiple task trainers may be created. For this price, 7–9 task trainers could be anticipated. After the mold is constructed or purchased, this price will decrease to $75 for subsequent creation of 7–9 task trainers.

### Detailed methods to construct this innovation

Instructions:

Silicone Part A:B

Ratio 1:1

1. Lay out Dragon Skin FX Pro- Part A and B, silicone pigment system, arm tubing, chest drain tubing, mold, mold release spray, rubber bands, tongue depressors, brush, clips, and cup before getting started. Wear gloves and ensure the room has proper ventilation.
2. Prepare penile mold with mold release spray (Image 2A). Our team used a purchased “bachelorette candle mold,” but this could be 3D printed as well. If mold is silicone based, petroleum jelly should also be used to line mold. Prepare a small amount of pigmented silicone (thoroughly mixing pigment into part B first and adding part A to that mixture) to brush on one side of the candle mold (Image 2B).
3. Allow the mixture to thicken and lay down chest drain tubing and peripheral intravenous (PIV) tubing for the silicone to bind to and hold in place (approximating urethra and corpus cavernosa). Repeat this process on the opposite mold as well, so you have created a pair with one piece of chest tube tubing in each.
4. While holding or securing the tubing in place, wait for silicone to mostly cure before prepping another batch of silicone to pour inside. This should take about 45 minutes.
5. After the tubing system is secured, assemble mold and wrap it with rubber bands. Place entire two-sided mold in cup, sitting upright. Pour a fresh batch of silicone into the remaining, empty spaces and allow it to cure for an additional 45 minutes (Image 2C and 2D).[Fig f3-jetem-11-4-i9]

### Results and tips for successful implementation

This session was initially implemented as a didactic improvement without data collection. Thereafter, we hosted a session, and at the end data was collected – specifically a post-session survey. Learners were asked questions about their confidence in and knowledge of the procedure as well as demographic data of their experience: how many times they had performed the procedure in the clinical setting and how successful the task trainer was in replicating the procedure. We have not currently modified the task trainer following implementation. We had 27 participants, including 14 EM residents, 5 pediatric EM fellows, 4 community EM attendings and 4 academic EM attendings. Overall, feedback was positive and reflected increased confidence in knowledge of the steps of the procedure as well as increased confidence to perform the procedure in the clinical setting. Participants overall would recommend the training to a provider of a similar level of training.

Once the prototype design was finalized, construction required approximately two hours (one hour hands-on and 1 hour curing time). This innovation has been used in five sessions with over 40 learners without meaningful degradation of the material. We do, however, anticipate that ultimately leaks will form and require either patching or replacement of task trainer.

A limitation of this study is that self-confidence was measured using self-reported data, which may be subject to response bias, social desirability effects, or inaccuracies in participants’ self-perceptions. Additionally, no rubric-based objective assessment of skills was conducted. As a result, changes in self-confidence could not be directly compared with objectively measured performance or competency, limiting the ability to determine whether increased confidence reflected actual skill development.[Table t2-jetem-11-4-i9][Fig f4-jetem-11-4-i9][Fig f5-jetem-11-4-i9]

**Counts/frequency:** 0 (12, 44.4%), 1 (6, 22.2%), 2 (5, 18.5%), 3–5 (3, 11.1%), 6–10 (0, 0.0%), >10 (1, 3.7%)

### Associated content

- Appendix A: Priapism Got You Down? PowerPoint

**Image 1 f1-jetem-11-4-i9:**
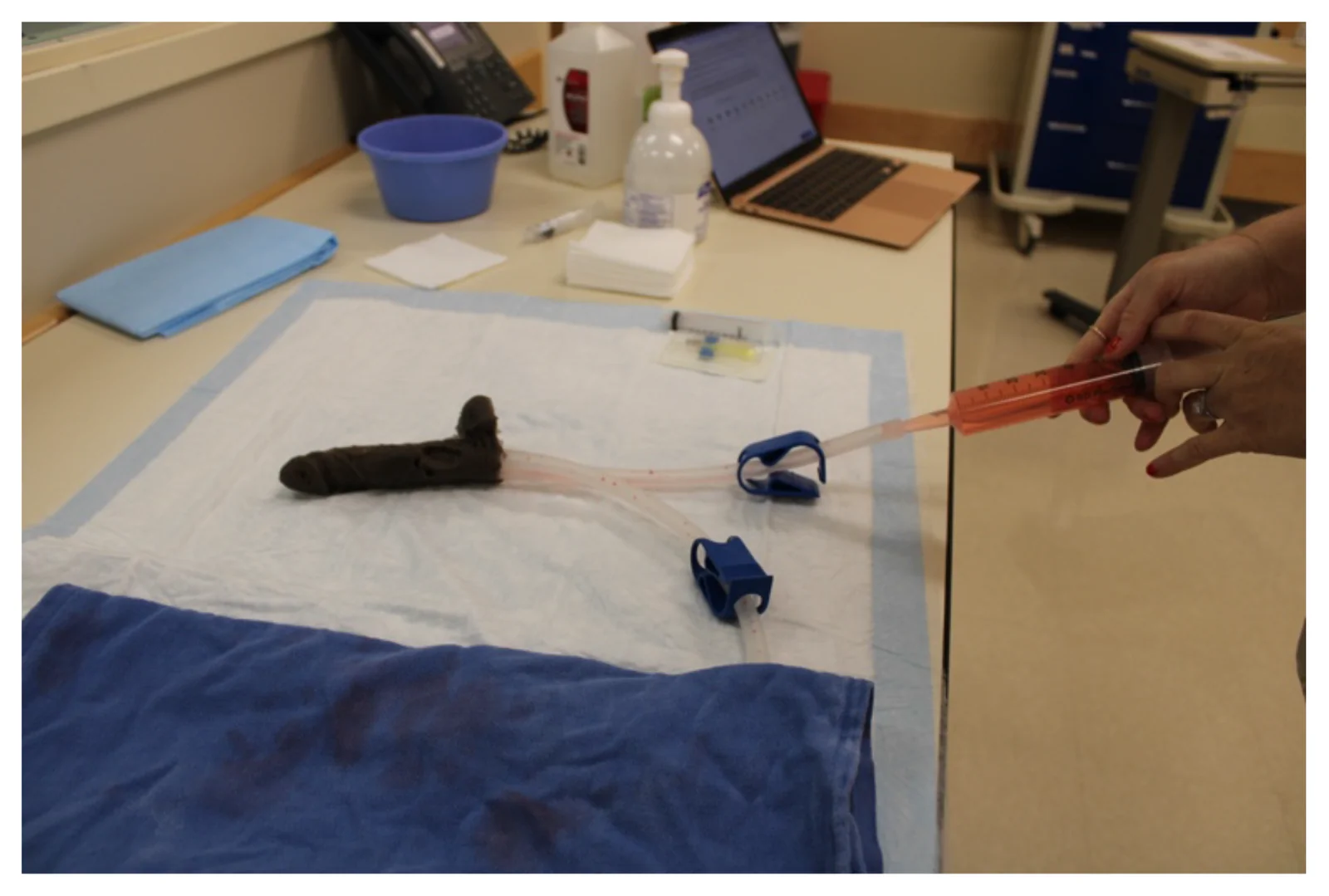
Task trainer being refilled with simulated blood: Facilitator is refilling unclamped tubing with simulated blood between sessions. After filling, the tubing will be clamped to hold it in place and the task trainer positioned anatomically with tubing hidden by drape until drained by participant.^9^

**Image 2A–D:**
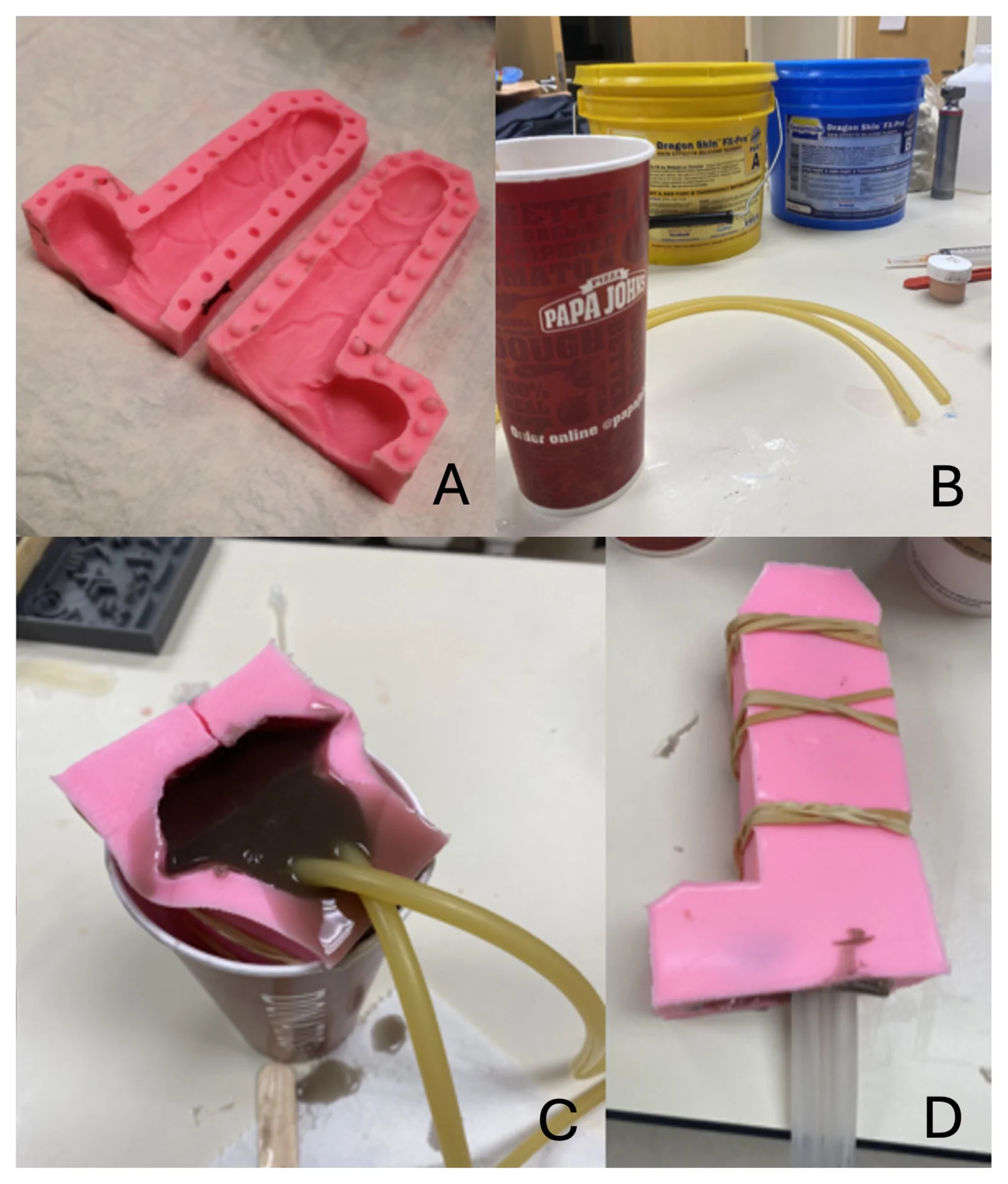
Supply layout: Pictures of supplies laid out and in process of task trainer creation.^10^

**Image 3 f3-jetem-11-4-i9:**
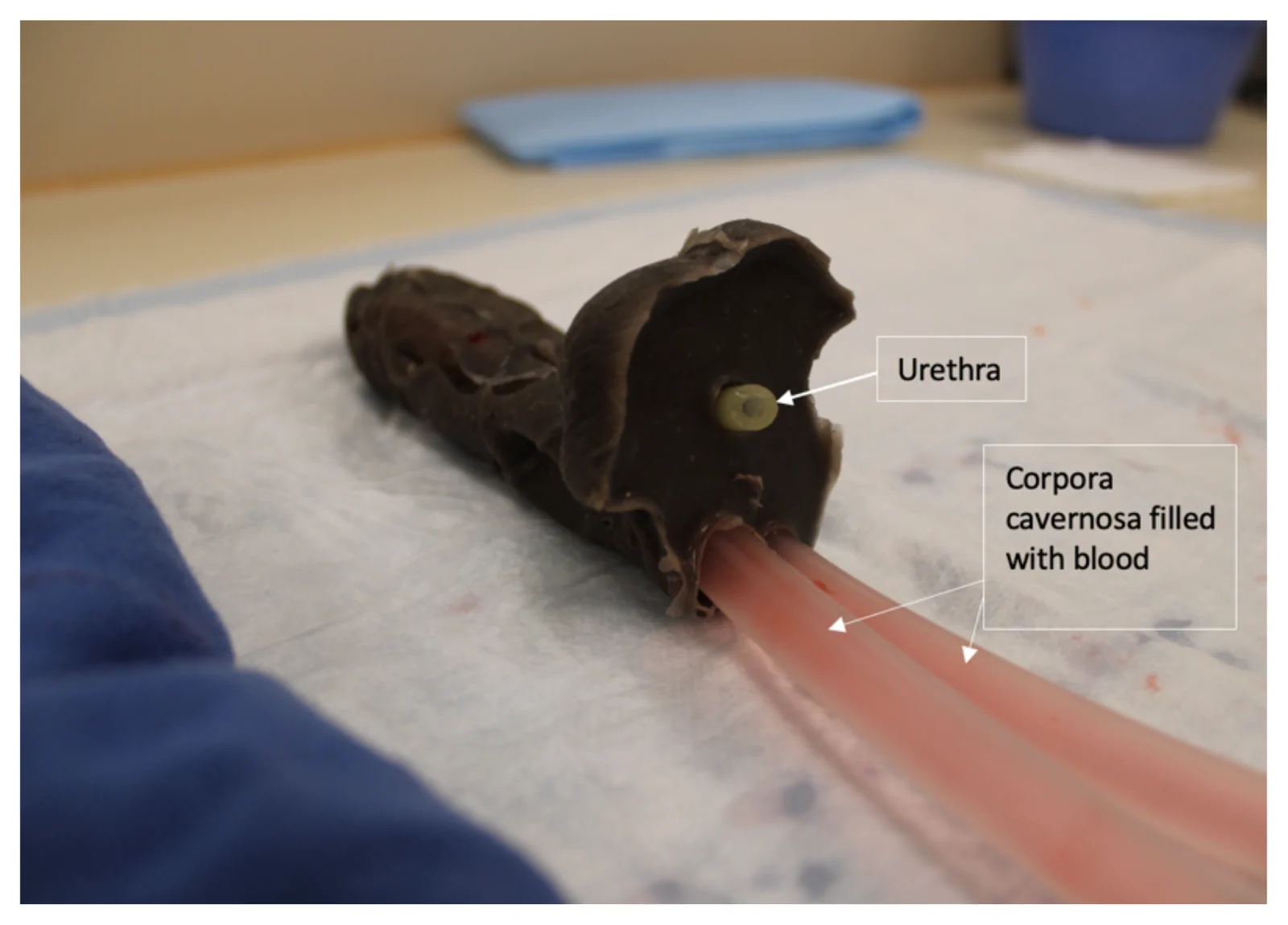
Task Trainer Tubing: Back of task trainer showing tubing inflow after refill.^11^

**Graph 1 f4-jetem-11-4-i9:**
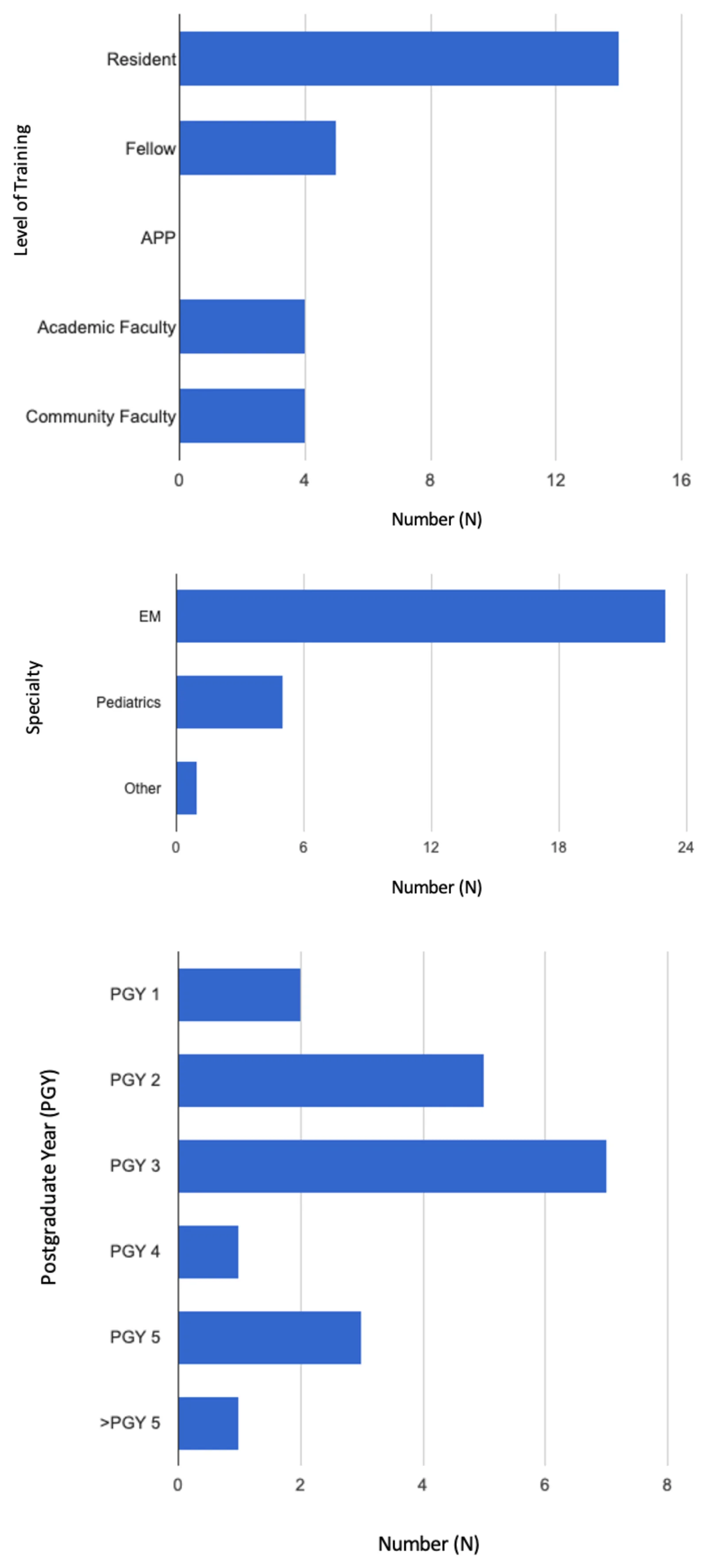
Demographics of learners by training level, specialty, and post-graduate (PGY) year: N=27.

**Graph 2 f5-jetem-11-4-i9:**
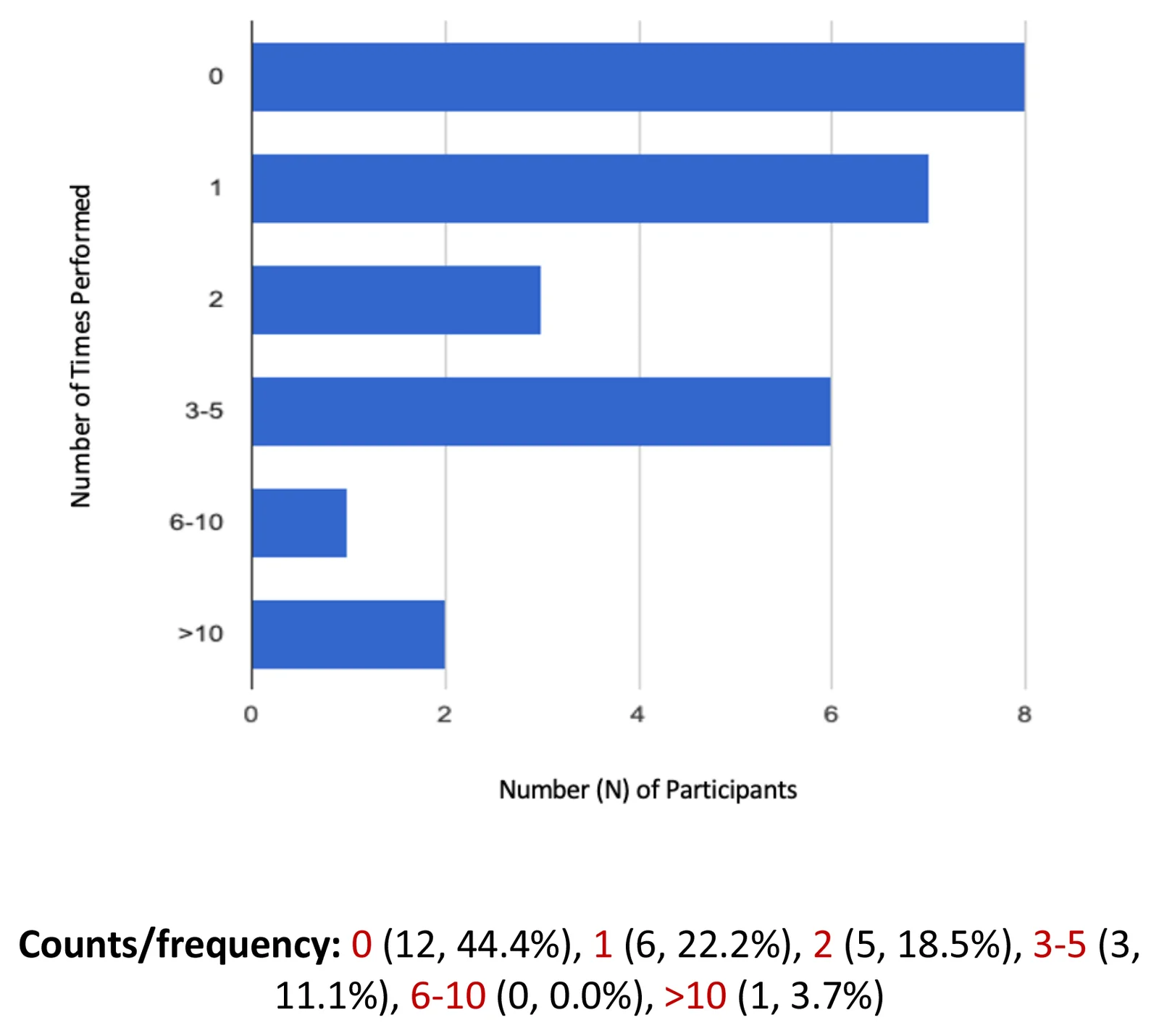
Survey results of participants self-reporting number of times they have aspirated for priapism in the clinical setting.

**Table 1:** Pre- and Post- Session Confidence For All Participants

Pre	Strongly Disagree	Disagree	Neutral	Agree	Strongly Agree
This procedure makes me anxious in the clinical environment.	0.0%	14.8%	37.0%	37.0%	11.1%
Before this training, I felt confident that I knew all of the steps to the procedure.	22.2%	25.9%	25.9%	25.9%	0.0%

Post	Strongly Disagree	Disagree	Neutral	Agree	Strongly Agree
Following this training, I feel confident that I know all of the steps to the procedure.	0.0%	0.0%	3.7%	70.4%	25.9%
Performing this procedure in the simulation setting increased my confidence to safely and successfully perform it clinically.	0.0%	0.0%	3.7%	48.1%	48.1%
Overall, I feel I could perform this procedure safely and successfully, unsupervised.	0.0%	0.0%	18.5%	55.6%	25.9%
I would recommend this training to colleagues of my same level of training.	0.0%	0.0%	3.7%	48.1%	48.1%

**Table/Image 2 t2-jetem-11-4-i9:** Survey results regarding task trainer fidelity (all participants)

	Strongly Disagree	Disagree	Neutral	Agree	Strongly Agree
This task trainer is realistic.	0.0%	3.7%	3.7%	70.4%	22.2%

## Supplementary Information



## Figures and Tables

**Figure:**
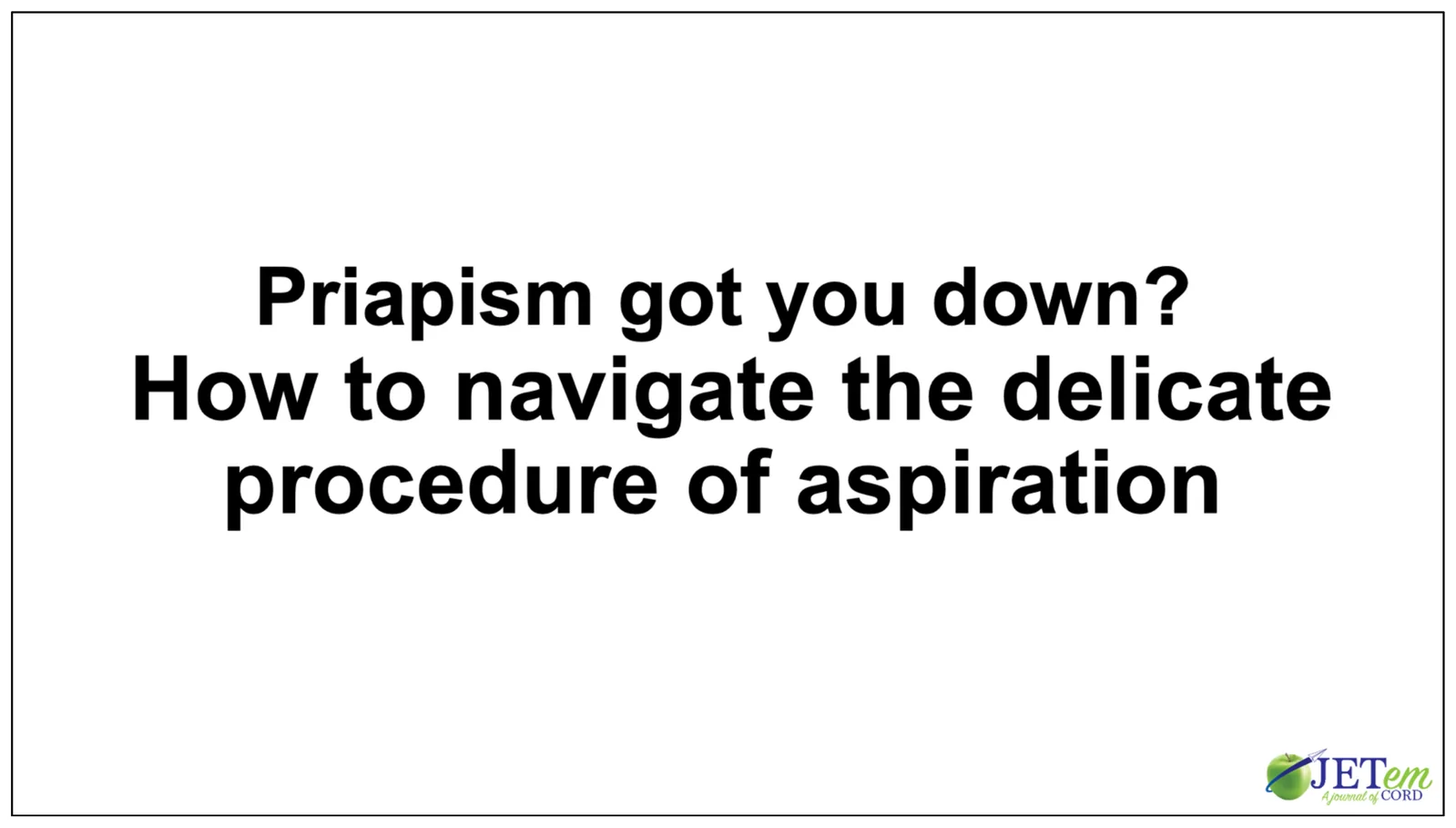
Please see associated PowerPoint file
